# Influence of vessel-depleted neck and risk factors on vascularized free flap failure: a retrospective cohort study and predictive model

**DOI:** 10.7717/peerj.21541

**Published:** 2026-07-22

**Authors:** Pingchuan Ma, Guile Zhao, Guanru Wang, Yufei Hua, Zhiyong Guo, Longjiang Li, Chunjie Li

**Affiliations:** State Key Laboratory of Oral Diseases & National Center for Stomatology & National Clinical Research Center for Oral Diseases & Department of Head and Neck Oncology, West China Hospital of Stomatology, Sichuan University, Chengdu, Sichuan, China

**Keywords:** Vascularized free flap transplantation, Head and neck reconstruction, Vessel-depleted neck, Free flap failure, Nomogram

## Abstract

**Background:**

The vessel-depleted neck represents a distinct clinical entity in free flap reconstruction, yet its impact on flap outcomes remains controversial. This study aimed to compare the rate of free flap failure between vessel-depleted neck cases and ordinary cases in head and neck reconstruction, and to identify risk factors for flap failure while developing a perioperative predictive model.

**Methods:**

We analyzed all vascularized free flap cases performed at our institution between July 2021 and June 2024. Cases were divided into training and testing sets based on surgery time. The association between vessel-depleted neck and flap failure was assessed. Multivariate logistic regression analysis was performed to identify independent risk factors for flap failure in the training set. A predictive model was subsequently constructed and validated using the testing set.

**Results:**

Among 1,965 free flap cases in analysis, 61 cases in the training set (*n* = 1, 506) were vessel-depleted neck cases. The incidence of flap failure in this subgroup was 4.9% (3/61), which was not significantly different from that in ordinary cases (*P* = 0.552). Multivariate logistic regression analysis revealed that liver disease (*P* = 0.001), maxillary disease location (*P* = 0.001), titanium plate use (*P* = 0.033), and surgical site infection (*P* < 0.001) were significant independent risk factors for flap failure. Details regarding recipient vessels were also recorded. A perioperative predictive model incorporating these four factors was developed and demonstrated an area under the curve (AUC) of 0.782 in the testing set.

**Conclusion:**

There was no significant difference in flap failure rates between vessel-depleted neck cases and ordinary cases. Liver disease, titanium plate use, maxillary disease location, and surgical site infection were identified as significant risk factors for flap failure. The developed predictive model demonstrated clinically useful discriminatory ability.

## Introduction

Advances in microsurgery have established vascularized free flap transplantation as the standard approach for reconstructing head and neck defects, playing a vital role in restoring postoperative function and improving patient quality of life (Corbitt et al., 2014; Wong & Wei, 2010). Over the past decades, the success rate of microsurgical procedures has steadily increased, consistently exceeding 95% in contemporary studies (Frederick et al., 2013; Yu et al., 2009). Although well-defined clinical protocols exist for free flap reconstruction in routine patients (Alhefzi et al., 2023; Yazar, 2007), management strategies in certain complex scenarios continue to pose challenges (Kota et al., 2024). One such challenging condition is the “vessel-depleted neck,” in which both recipient vessel selection and flap success remain subjects of ongoing debate.

The definition and clinical scope of the “vessel-depleted neck” remain inconsistent across studies (Martinez et al., 2020; Tsao, Loh & Barrera, 2016; Wong, Hung & Wei, 2025). In general, the term refers to patients who have undergone prior neck dissection, with or without adjuvant radiotherapy. Regardless of the specific definition applied, the principal reconstructive challenge lies in identifying and preparing suitable recipient vessels for microsurgical anastomosis, a difficulty frequently encountered in head and neck reconstruction (Mourad et al., 2015). Distinct from the “frozen neck”, which primarily refers to severe post-radiation fibrosis with or without prior surgery (Shih et al., 2013; Yu, 2005), the vessel-depleted neck specifically denotes a condition with limited recipient vessels following neck dissection, typically without significant soft tissue deficiency. Theoretically, compromised vascular conditions may increase the technical difficulty of microsurgical reconstruction and potentially elevate the risk of flap failure. However, clinical evidence supporting this association remains limited (Nahabedian et al., 2004). Some previous studies did not clearly document the extent of prior neck dissection within their cohorts (Ooms et al., 2023), whereas others primarily reported flap outcomes in vessel-depleted neck patients without comparison to ordinary cases or comprehensive multivariable analysis (Head et al., 2002; Kadota et al., 2012). Consequently, further high-quality evidence and rigorous analysis are still needed to better characterize free flap outcomes in well-defined vessel-depleted neck cases.

Over the past few decades, studies have sought to identify potential risk factors for failure in vascularized free flap reconstruction of head and neck defects. Possible related factors investigated include prolonged hospitalization (Mücke et al., 2016), hypertension (Qiao et al., 2022) and chemotherapy (Chang et al., 2016; Lambert et al., 2023). Other variables such as advanced age or smoking were not consistently associated with flap survival in some studies (Spindler et al., 2021; Wong et al., 2015). However, a notable limitation of the existing literature is its predominant focus on a narrow set of general risk factors, often omitting critical microsurgery-specific technical variables-such as anastomotic techniques or recipient vessel selection strategies. Moreover, efforts to develop and validate predictive models for free flap failure in head and neck reconstruction based on established risk factors also remain scarce. Therefore, the present study aimed to: (1) comprehensively compare flap failure rates between vessel-depleted neck cases and ordinary cases; (2) summarize optimal recipient vessel selection strategies in this challenging context; and (3) identify both patient-related and surgery-related factors influencing flap success, and subsequently develop a predictive model for estimating the risk of free flap failure in head and neck reconstruction.

## Materials & Methods

### Study design

This cohort study included free flap reconstruction surgery cases for head and neck diseases in the Department of Head and Neck Oncology, West China Hospital of Stomatology, Sichuan University between July 2021 and June 2024 and was approved by the institutional review board of West China Hospital of Stomatology (Ethical approval number: WCHSIRB-D-2024-185). Written informed consents were obtained from patients. The study was conducted and reported in accordance with the Strengthening the Reporting of Observational Studies in Epidemiology (STROBE) statement. We retrospectively collected the cases between July 2021 and December 2023 as training set, and prospectively enrolled the cases since January 2024 to June 2024 to constitute testing set. The inclusion criteria were as follows: (1) All vascularized free flap procedures performed for head and neck reconstruction at West China Hospital of Stomatology, Sichuan University during the study period. (2) Patient age between 10 and 95 years. The exclusion criteria were: (1) For patients who underwent an unplanned second free flap reconstruction with debridement during their treatment, the second free flap surgeries were excluded, while the initial free flap procedure was still included in the analysis and classified as flap failure. (2) The cases with incomplete clinical data were excluded. Among included cases, a vessel-depleted neck case was defined as one in which the patient had undergone prior neck dissection extending beyond level I on the same side as the current free flap surgery, with or without radiotherapy, and the commonly used recipient vessels were sacrificed or unsuitable for anastomosis.

After case inclusion, baseline data were collected and characteristics were summarized in both sets. An analysis was first performed in the training set to compare the rates of postoperative complications (including flap status, surgical site infection, and unplanned reoperation) between vessel-depleted neck cases and ordinary cases. After that, the univariate and multivariate analyses were applied in the training set to identify the potential risk factors for free flap failure. Using the identified risk factors, a predictive model for free flap failure was developed and the model was then evaluated using the testing set. In this study, several approaches were implemented to minimize potential bias. Cases were consecutively enrolled according to criteria and parameters were accurately recorded. Multivariate regression was performed to adjust for confounding as mentioned above.

### Data collection

The medical records of included cases were systematically reviewed. The following parameters were recorded: age, gender, smoking history, alcohol intake history, diabetes mellitus, hypertension, heart disease, liver disease, radiotherapy history, chemotherapy history, hospitalization history, operation history, recurrence, operation duration, blood loss, tracheotomy, titanium plate use, disease location, disease types, histopathology, flap types, surgical site infection, unplanned reoperation and recipient vessels. We referred to the guidelines updated by the CDC in 2017 as the diagnostic criteria for surgical site infection (SSI) (Berrios-Torres et al., 2017).

### Statistical analysis and logistic regression

Statistical analyses were performed using SPSS Statistics version 26.0 (IBM Corp., Armonk, NY, USA). Patient characteristics were presented as means with standard deviations for continuous variables and as frequencies with percentages for categorical variables. Differences in continuous variables were assessed using the Wilcoxon rank-sum test, while categorical variables were compared using Pearson’s chi-squared test or Fisher’s exact test, as appropriate. Univariate logistic regression analysis was initially performed to evaluate the associations between individual variables and flap failure. Multivariate logistic regression analyses were performed using both the Enter and Forward: LR (forward stepwise based on likelihood ratios) models. Forest plots were used to visualize the results of both univariate and multivariate analyses and a *P* value < 0.05 was considered statistically significant. All plots were generated using GraphPad Prism version 8, and figures were prepared with Adobe Illustrator 2020.

### Predictive model development

Independent risk factors that demonstrated significance in both of the two multivariate logistic regression models were incorporated into a nomogram constructed using the “rms” package in R (version 4.2). The nomogram was developed based on the regression coefficients (β values) derived from the Forward: LR model multivariate logistic regression. Each predictor was assigned a point value proportional to its β coefficient relative to the predictor with the largest absolute β value, which was scaled to 100 points. The total score for an individual patient was obtained by summing the points assigned to each predictor. The predicted probability of free flap failure was calculated according to the logistic regression function:

\begin{eqnarray*}P= \frac{1}{1+{e}^{-({\mathrm{\beta }}_{0}+\sum {\mathrm{\beta }}_{i}{X}_{i})}} \end{eqnarray*}

where β0 represents the intercept and βi represents the regression coefficient corresponding to predictor Xi. For clinical application, each predictor value is first located on its respective axis in the nomogram to determine the assigned score. The individual scores are then summed to generate a total score, which corresponds to an estimated probability of free flap failure on the risk scale. Higher total scores indicate a greater predicted risk of flap failure. The model’s discriminative performance was evaluated in the independent testing set using receiver operating characteristic (ROC) curve analysis, and the area under the curve (AUC) was calculated to quantify predictive accuracy.

## Results

### Characteristics of the study cohort

**Figure 1:**
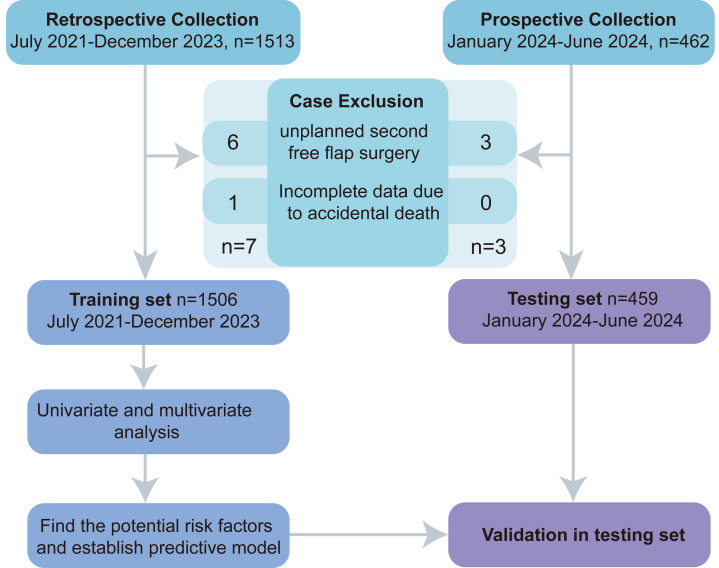
The flowchart of the cohort study design.

**Figure 2:**
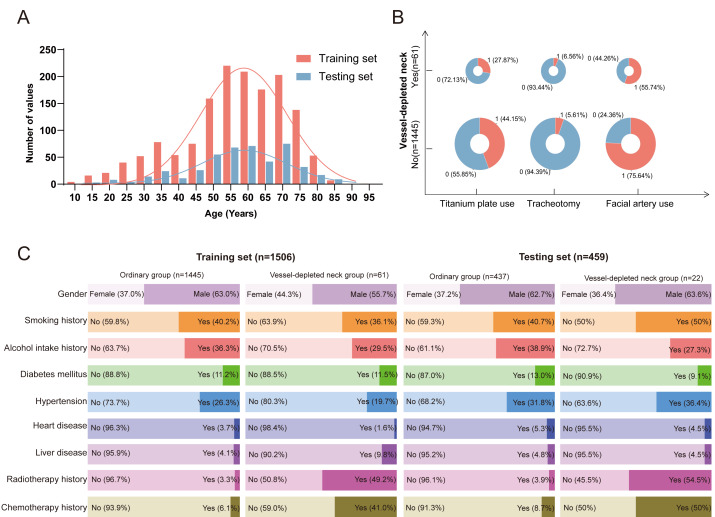
Characteristics of cases included in training set and testing set. (A) Age distribution and corresponding Gaussian fitted curves of patients in two sets. (B) Distribution of three operation related parameters (Titanium plate use, Tracheotomy and Facial artery use) in training set divided by vessel-depleted neck status (0 represents no and 1 represents yes). (C) Distribution of demographic characteristics in training set and testing set divided by vessel-depleted neck status.

A total of 1,975 free flap procedures for head and neck reconstruction were performed during the study period. After excluding 10 cases, 1,965 free flap surgeries were included in the final analysis. Retrospectively collected cases performed between July 2021 and December 2023 were assigned to the training set (*n* = 1,506), while prospectively collected cases performed between January and June 2024 comprised the testing set (*n* = 459). There were 83 cases classified as vessel-depleted neck status. The number of vessel-depleted neck cases in training set and testing set was 61 and 22. All cases in the vessel-depleted neck group had undergone prior surgical procedures, and 42 of them had also received radiotherapy. Overall study process is illustrated in Fig. 1 *via* a flowchart. The typical surgical procedure is shown (Fig. S1). Basic characteristics of cases in both sets were summarized in Fig. 2, Tables S1 and S2. In the training set, the mean age of patients in the ordinary group was 56.46 years, compared to 56.52 years in the vessel-depleted neck group. There were no statistically significant differences in baseline characteristics or systemic diseases between the two groups (*P* > 0.05). However, the vessel-depleted neck group had a higher proportion of patients with a history of radiotherapy (30/61), chemotherapy (25/61), and disease recurrence (39/61), which aligns with the condition for vessel-depleted neck cases. In the testing set, the mean age of patients in the ordinary and vessel-depleted neck groups was 57.56 and 59.09 years, respectively, which was consistent with the training set. The age distribution in both sets was also comparable. Similarly, no significant differences were observed in baseline characteristics or systemic disease between the two groups in the testing set.

### Association between vessel-depleted neck and flap failure

**Figure 3:**
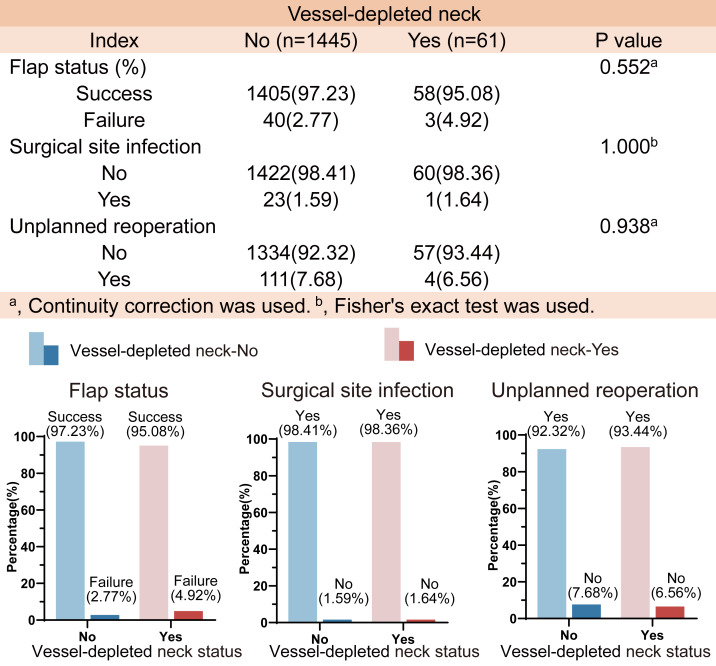
Association between vessel-depleted neck status and postoperative outcomes based on training set (Flap status, Surgical site infection and Unplanned reoperation). The blue vertical bars represent the data of ordinary cases and the red vertical bars represent the data of vessel-depleted neck cases.

We further explored the association between vessel-depleted neck status and postoperative complications including flap status, surgical site infection and unplanned reoperation in training set. According to our results, the incidence of flap failure in vessel-depleted neck cases was 4.9% (3/61), which was not significantly different from that in the ordinary group (*P* = 0.552; Fig. 3). Similarly, no significant differences were observed in the incidence of surgical site infection (*P* = 1.000) or unplanned reoperation (*P* = 0.938) between the two groups. These findings suggest that vessel-depleted neck cases do not confer a significantly higher risk of flap failure, surgical site infection, or unplanned reoperation than ordinary cases.

### Univariate analysis of factors influencing flap failure

We subsequently conducted univariate analyses incorporating both patient-related and surgery-related factors to illustrate potential risk factors associated with free flap failure in the training set. Among the patient-related factors, disease type (OR = 0.488; 95% CI [0.242–0.982]; *P* = 0.044) and age (OR = 0.976; 95% CI [0.959–0.995]; *P* = 0.011) were significantly associated with a reduced risk of flap failure (Table S3). In particular, patients with liver disease (OR = 3.859; 95% CI [1.567–9.501]; *P* = 0.003) and those with maxillary disease (OR = 6.955; 95% CI [2.177–22.215]; *P* = 0.001) were at higher risk of flap failure. Among the surgery-related factors, the use of titanium plates (OR = 2.244; 95% CI [1.199–4.200]; *P* = 0.012) and the occurrence of surgical site infection (OR = 7.400; 95% CI [2.415–22.672]; *P* < 0.001) were significantly associated with increased flap failure (Table S4). Other evaluated factors did not reach statistical significance in the univariate analysis.

### Multivariate analysis of factors influencing flap failure

**Figure 4:**
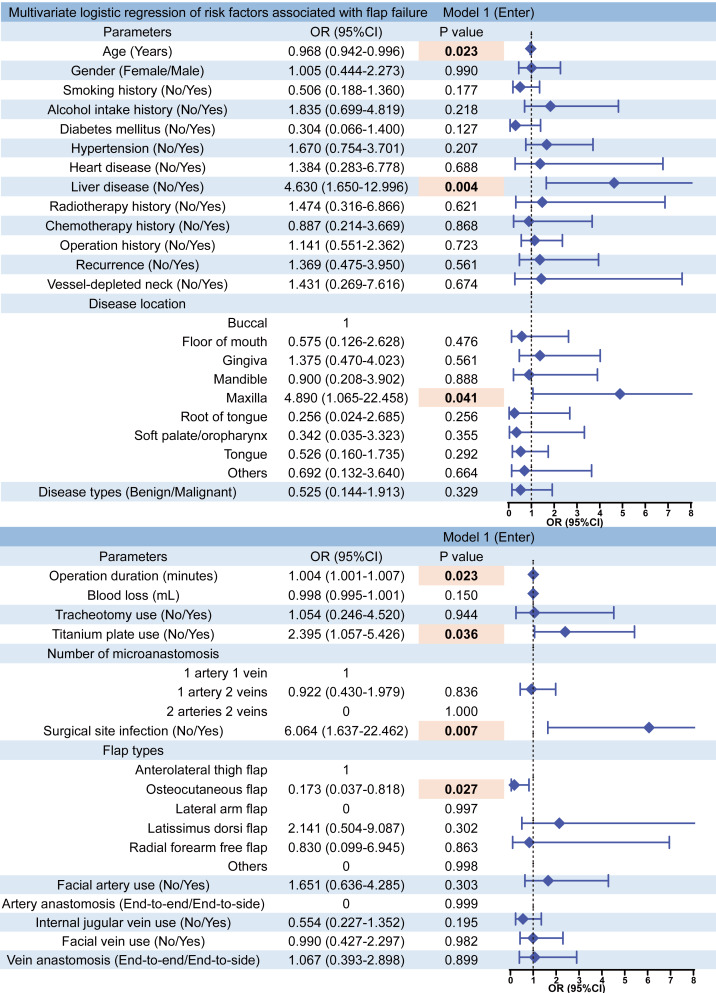
Multivariate logistic regression analysis for risk factors associated with flap failure (Enter model) and the corresponding forest plot. Bolded values indicate statistical significance (OR: odds ratio; CI: confidence interval).

**Figure 5:**
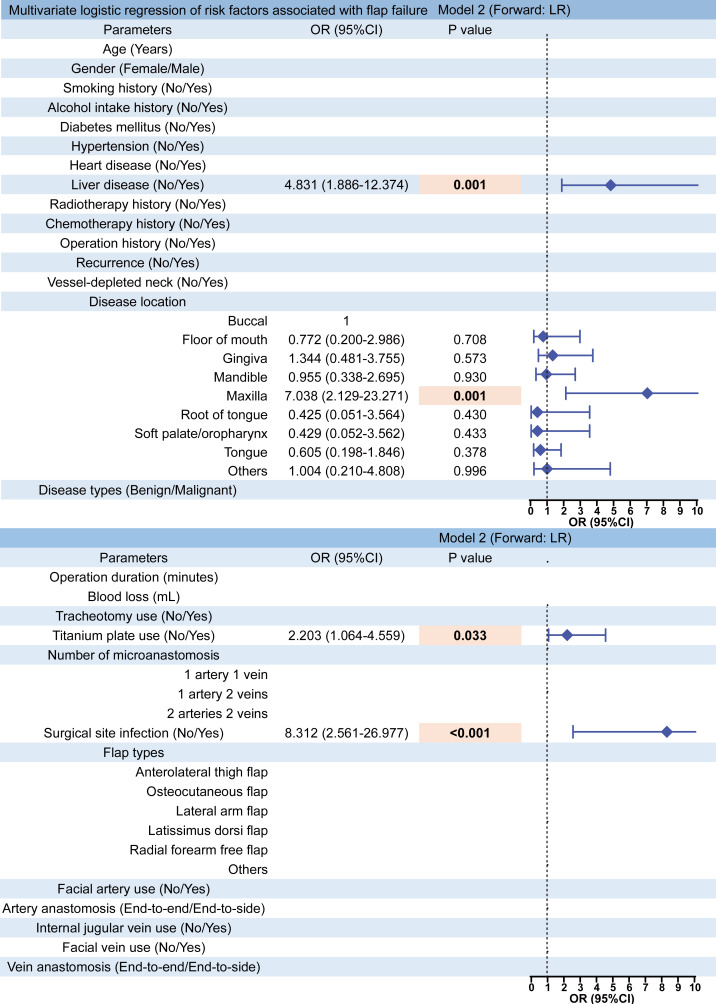
Multivariate logistic regression analysis for risk factors associated with flap failure (Forward: LR model) and the corresponding forest plot. Bolded values indicate statistical significance (OR: odds ratio; CI: confidence interval).

Following the univariate analysis, we performed multivariate analyses using both Enter and Forward: LR models. The results of the multivariate analysis are presented in Figs. 4 and 5. In the Enter model, age remained significantly associated with flap failure (OR = 0.968, 95% CI [0.942–0.996], *P* = 0.023). Additionally, several factors were also associated with a significantly higher incidence of flap failure: liver disease (OR = 4.630, 95% CI [1.650–12.996], *P* = 0.004), maxilla disease location (OR = 4.890, 95% CI [1.065–22.458], *P* = 0.041), titanium plate use (OR = 2.395, 95% CI [1.057–5.426], *P* = 0.036), longer operation duration (OR = 1.004, 95% CI [1.001–1.007], *P* = 0.023), and surgical site infection (OR = 6.064, 95% CI [1.637–22.462], *P* = 0.007). In the Forward: LR model (Fig. 5), significant risk factors included liver disease (OR = 4.831, 95% CI [1.886–12.374], *P* = 0.001), maxilla disease location (OR = 7.038, 95% CI [2.129–23.271], *P* = 0.001), titanium plate use (OR = 2.203, 95% CI [1.064–4.559], *P* = 0.033), and surgical site infection (OR = 8.312, 95% CI [2.561–26.977], *P* < 0.001). No other factors demonstrated a significant impact on free flap failure in our analyses.

Finally, we summarized the recipient arteries utilized in vessel-depleted neck cases across both the training and testing sets (Table S5). In the training set, the contralateral facial artery was the most frequently used recipient vessel, accounting for over half of the cases (33/61), and was also the most common choice in the testing set (9/22). Beyond the contralateral vessels, the ipsilateral superficial temporal artery (12/83) and superior thyroid artery (11/83) were both reliable recipient vessel options. Notably, there was no flap failure in the testing set vessel-depleted neck cases. Within the training set, the three flap failures occurred in cases utilizing the contralateral facial artery (*n* = 2) and the ipsilateral superficial temporal artery (*n* = 1). It should be noted that cases utilizing vein grafting (*n* = 1) or flow-through flaps (*n* = 2) were exceptionally rare within our vessel-depleted group so we did not include these factors in analysis. Flap reconstruction was successful in these cases, utilizing the ipsilateral and contralateral superior thyroid arteries as recipient arteries. The recipient vein selected were internal jugular vein and external jugular vein.

### Establishment and verification of predictive model

**Figure 6:**
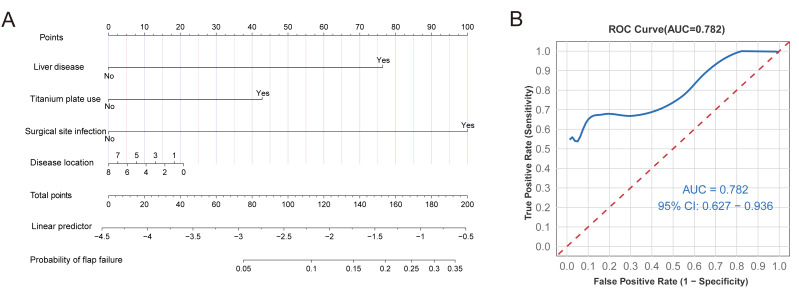
Nomogram predicting free flap failure and its validation in the testing set. (A) Nomogram for free flap failure prediction incorporating four factors (Disease location: 0 = Maxilla, 1 = Gingiva, 2 = Mandible, 3 = Buccal, 4 = Floor of mouth, 5 = Others, 6 = Root of tongue, 7 = Soft palate/oropharynx, 8 = Tongue). (B) The receiver operating characteristic (ROC) curve analysis showing the nomogram’s performance in the testing set (AUC: area under the curve).

Based on the four independent predictors that demonstrated significance in both multivariate logistic regression analyses: liver disease, disease location, titanium plate use, and surgical site infection, we constructed a predictive nomogram (Fig. 6). These four factors were selected because they remained statistically significant in both the Enter and Forward: LR multivariate regression models. For an individual case, each predictor was assigned a point value proportional to its β coefficient relative to the predictor with the largest absolute β value, which was scaled to 100 points. These scores are summed to yield a total point score. A higher total score correlates with an increased probability of free flap failure. We subsequently validated this predictive model in the testing set (*n* = 459). Receiver operating characteristic (ROC) curve analysis demonstrated good discriminatory ability, with an area under the curve (AUC) of 0.782 (95% CI [0.627–0.936]) (Fig. 6). The model in this study could calculate a total score by summing the corresponding scores of the four factors. And the probability of free flap failure is subsequently determined on the basis of the total score.

## Discussion

The vessel-depleted neck represents a clinically challenging condition in head and neck free flap reconstruction. This study performed a comparative analysis of free flap outcomes between vessel-depleted neck cases and ordinary cases. We also incorporated this factor into multivariate analysis alongside other microsurgical related factors and developed a predictive model. All analyses were performed in a large cohort consisting of a retrospective training set and a prospective testing set.

We first compared postoperative outcomes between vessel-depleted neck cases and ordinary cases, focusing on free flap survival, surgical site infection, and unplanned reoperation. Our results indicated that although surgical complexity may be greater in vessel-depleted neck cases, the free flap failure rate was not significantly increased. This finding is consistent with previous studies. Head et al. (2002) reviewed 60 head and neck free flap reconstructions in patients with prior neck dissection and reported no flap failures. More than half of their cases utilized contralateral recipient arteries for anastomosis, which was also consistent with our findings. However, their study mainly reported flap success rates without comprehensive analysis of related clinical variables. Similarly, Tan et al. (2014) investigated free flap reconstruction following neck dissection and reported a flap failure rate of 5.9% (5/84) in the prior neck dissection group, with no significant difference compared with control groups. Collectively, these studies suggest that free flap failure rates remain low despite the technical challenges associated with limited recipient vessel availability and complex microvascular anastomosis, which is in accordance with our results. In our training set, the flap failure rate in vessel-depleted neck cases was 4.9% (3/61, *P* = 0.552), which was not significantly different from that in ordinary cases. This outcome is consistent with our clinical experience (Wu et al., 2021), in which adequate flap pedicle length has generally allowed reliable contralateral microvascular anastomosis. Furthermore, our analysis revealed no significant association between vessel-depleted neck status and surgical site infection or unplanned reoperation. These findings collectively indicated that vascularized free flap reconstruction in the head and neck region remains a highly successful procedure for patients with a history of neck dissection. The incidence of postoperative complications does not appear to increase in this population when employing appropriate surgical strategies, perioperative management, and rational antibiotic use.

We further performed univariate and multivariate analyses to identify factors potentially associated with free flap failure. Previous studies have explored various clinical risk factors. Mücke et al. (2016) analyzed 451 patients (4% failure rate) and identified prior attempts at microvascular transplantation and prolonged hospitalization as risk factors for flap failure. Zhou et al. (2017) reported that previous irradiation significantly increased flap failure risk in their cohort of 881 cases (2.9% failure rate). In our study, liver disease, titanium plate use, and surgical site infection were identified as potential risk factors for flap failure. Although systemic disease has previously been associated with flap outcomes, evidence regarding liver disease remains limited (Rosado et al., 2015). Callander et al. (2022) reported that hepatitis C virus (HCV) positivity did not correlate with flap failure in head and neck reconstruction. Given limited evidence, we speculate that liver disease (*e.g.*, cirrhosis, viral hepatitis) may affect flap survival through impaired coagulation and hemodynamic instability, although this relationship requires further investigation. In addition, there is currently no conclusive evidence linking flap failure to the use of titanium plates; however, a plausible explanation for this observed trend can be offered. In patients with extensive mandibular involvement or advanced disease, most cases require either segmental or marginal mandibulectomy, followed by the use of titanium plates. These patients typically present with greater surgical complexity, more extensive tissue compromise, and a higher systemic and oncologic burden, all of which may contribute to increased difficulty in free flap transplantation. Thus, this association between titanium plate use and flap failure likely reflects unmeasured complexity in reconstructions rather than a direct biological effect of the plates themselves. Meanwhile, we noticed a discrepancy in the Enter model multivariate analysis: titanium plate use was associated with increased flap failure, whereas osteocutaneous flaps showed a seemingly protective effect. It seems contradictory as osteocutaneous flaps generally use titanium plates in surgery. This finding should be interpreted with caution. The protective association of osteocutaneous flaps was not consistently observed across univariate and multivariate models, suggesting that it may not represent a stable independent predictor. Furthermore, this discrepancy may be explained by differences in case selection and reconstructive strategy. In our clinical practice, osteocutaneous flaps are typically used in patients undergoing planned bony reconstruction under relatively controlled conditions, which may reflect more favorable baseline status and surgical planning. In addition to these patients, titanium plates are also frequently used in cancer patients with extensive mandibular involvement, advanced disease, or complex composite defects. For these cases, an extensive segmental or marginal mandibulectomy was involved with titanium plates used and technically less demanding soft tissue free flaps were often selected. These cases generally involve greater surgical complexity, compromised local tissue conditions, and higher systemic or oncologic burden, which may increase the risk of flap failure. Therefore, titanium plate use may act as a surrogate marker of reconstruction complexity rather than a direct causal factor for flap failure. Another important finding was the association between surgical site infection and flap failure. Although infected patients showed a higher flap failure rate, potential reverse causality should be considered, as flap compromise itself may contribute to postoperative infection. Las et al. (2016) identified postoperative wound infection as a risk factor in extremity reconstruction but not in head and neck reconstruction. Therefore, while an association was observed in our study, the causal relationship between surgical site infection and flap failure remains unclear and requires further investigation. In addition, maxillary disease location was identified as a risk factor was also difficult to accurately explain as studies have confirmed the high successful rate (Cho et al., 2025). We speculate that the greater distance between maxillary defects and cervical recipient vessels may increase surgical complexity in these cases.

As this was a cohort study without predefined group allocation and vessel-depleted neck cases are relatively uncommon, a substantial imbalance existed between the vessel-depleted and ordinary groups. To reduce potential confounding and minimize the risk of model overfitting, multivariate regression with careful variable selection was performed. Based on the multivariate analysis results, four risk factors, which were identified as statistically significant in both models, were incorporated into a predictive model. Predictive modeling remains uncommon in the free flap outcome literature. Liu et al. (2024) previously reported a high-performing model for fibular free flap necrosis in mandibular osteoradionecrosis; however, their study focused specifically on fibular flaps. In contrast, our model was designed for overall head and neck free flap reconstruction. We identified liver disease, titanium plate use, surgical site infection, and maxillary disease location as negative predictors of flap survival; but the resulting AUC of 0.782 was lower than Liu’s reported performance. This suggests that more accurate prediction of free flap failure requires the identification of additional significant variables and further model improvement. Even so, we believe our predictive model still offers valuable guidance for clinical practice. The proposed model can serve as a perioperative and early postoperative risk prediction tool for the entire treatment process of vascularized free flap patients. It enables clinicians to dynamically evaluate the risk of flap failure as new clinical data become available, particularly once early postoperative variables are known, and to adapt their management plans accordingly. The development of flap compromise is not determined solely by preoperative factors, but is also influenced by intraoperative conditions and postoperative management. Preoperatively, in patients presenting with identified risk factors or a high predicted probability of flap, extra caution should be exercised when opting for free flap reconstruction, and alternative reconstructive options should be carefully considered. And if a patient shows signs of surgical site infection postoperatively, this serves as an indicator to doctors for closer flap monitoring and prompt intervention to mitigate infection and support flap survival, while maintaining readiness for potential flap salvage procedures. Therefore, the model is intended to support ongoing clinical decision-making, rather than solely preoperative counselling and may be helpful in real clinical situations.

Previous studies have addressed surgical management and recipient vessel selection in both frozen and vessel-depleted necks (Chang et al., 2023; Navarro Cuellar et al., 2021; Sudirman et al., 2019). In alignment with prior reports (Kushida-Contreras, Manrique & Gaxiola-García, 2021; Reddy et al., 2020), contralateral vessels proved reliable for microvascular anastomosis in our study. The contralateral facial artery was utilized in approximately half of cases (42/83) with only two failures. This finding supports that, given adequate pedicle length, the contralateral facial or superior thyroid arteries constitute viable options in vessel-depleted neck cases. Regarding ipsilateral options, given the frequent sacrifice of the facial artery during previous neck dissections, alternative arteries remain feasible. The ipsilateral superficial temporal artery (12/83) and superior thyroid artery (11/83) were most frequently employed (Frohwitter et al., 2018; Tessler et al., 2017). In special scenarios, anastomosis to prior free flap arteries or the ipsilateral external carotid artery (end-to-side) also proved feasible according to our results. Collectively, these findings underscore the critical importance of preoperative vascular assessment and contingency planning for microvascular anastomosis (Martinez et al., 2020).

Several limitations of this study should be acknowledged. First, as this was a cohort study, an imbalance in sample size existed between ordinary cases and vessel-depleted neck cases, with fewer patients in the latter group. This imbalance may have reduced the statistical power to detect group differences. Rather than performing matched analyses that would have excluded a substantial number of eligible cases, we retained all patients to preserve real clinical representativeness. Meanwhile, the relatively low number of flap failure events may introduce a potential risk of model overfitting and instability during model construction. To mitigate these issues, we applied multivariate logistic regression to conduct careful variable selection for the predictive model to reduce model complexity, and further evaluated the model using an independent testing set, which helped improve the assessment of model generalizability and reduce the likelihood of substantial overfitting. Second, the criteria used to define the vessel-depleted neck were relatively broad. The criteria were selected to reflect the real-world reconstructive challenges encountered in our clinical setting and were aligned with prior studies (Reddy et al., 2020). In our cohort, the proportion of vessel-depleted neck cases and the observed flap failure rates were within the ranges reported in previous literature, supporting the clinical reasonableness of this pragmatic definition. Given that extremely complex reconstruction cases are rare and that related case reports have documented specialized free flap approaches (Broderick, Vassiliou & Kyzas, 2021; Delay et al., 2023), the findings of this study may be more generalizable to real clinical practice within our regional and institutional context. While this broader pragmatic definition improves real-world applicability, it may also encompass patients with varying degrees of recipient vessel compromise and reconstructive difficulty, thereby introducing heterogeneity within the vessel-depleted neck cohort. Therefore, the findings should be interpreted with particular caution in contexts involving differing recipient vessel strategies or more strictly defined patient subsets, especially when compared with studies using more restrictive criteria. Third, we additionally evaluated the calibration and overall predictive performance of the model. Bootstrap-corrected calibration analysis in the training set demonstrated acceptable agreement between predicted and observed probabilities of flap failure. However, calibration performance in the testing set appeared less robust. This finding should be interpreted cautiously, as flap failure was a relatively infrequent event and the number of outcome events in the testing cohort was limited, which may have reduced the stability of calibration assessment and parameter estimation. Therefore, further validation in larger independent cohorts is still required to more comprehensively evaluate the external calibration performance and generalizability of the model.

## Conclusions

In this cohort study, there was no statistically significant difference in the rate of free flap failure in head and neck reconstruction between vessel-depleted neck cases and ordinary cases. Multivariate analysis identified liver disease, titanium plate use, maxillary disease location and surgical site infection as risk factors for flap failure. A predictive model incorporating these variables enables quantification of flap failure probability. Regarding recipient vessel selection in vessel-depleted neck cases, the contralateral facial artery, ipsilateral superficial temporal artery, and superior thyroid artery were the most commonly utilized options. When combined with proficient microsurgical technique, optimized surgical planning, and evidence-based perioperative management, stable success rates are achievable in this complex condition.

##  Supplemental Information

10.7717/peerj.21541/supp-1 Supplemental Information 1 Representative surgical procedure in a vessel-depleted neck patients in our study The presented patient was diagnosed as recurrence squamous cell carcinoma in tongue and has received left radical neck dissection with left anterior lateral thigh flap transplantation five years ago. This time the surgery plan was to use the right anterior medial thigh flap for reconstruction. (A) The initial exposure of left neck region. The left internal jugular vein was indicated (white arrow). (B) Resection of recurrent tongue lesion. (C) Microsurgical identification of recipient vessels: ipsilateral superior thyroid artery (white arrow) and internal jugular vein branch (black arrow). (D) End-to-end arterial anastomosis between superior thyroid artery and flap pedicle (white arrow). The venous anastomosis was applied between internal jugular vein branch and flap vein (black arrow).

10.7717/peerj.21541/supp-2 Supplemental Information 2 Characteristics of patients in training set based on vessel-depleted neck status

10.7717/peerj.21541/supp-3 Supplemental Information 3 Characteristics of patients in testing set based on vessel-depleted neck status

10.7717/peerj.21541/supp-4 Supplemental Information 4 Univariate logistic regression of patient factors associated with flap failure in training set

10.7717/peerj.21541/supp-5 Supplemental Information 5 Univariate logistic regression of surgical factors associated with flap failure in training set

10.7717/peerj.21541/supp-6 Supplemental Information 6 Arteries selection of vessel-depleted neck cases in two sets

10.7717/peerj.21541/supp-7 Supplemental Information 7 Raw Data All the clinical records of the included cases used in our analyses.

10.7717/peerj.21541/supp-8 Supplemental Information 8 Codebook of raw data

10.7717/peerj.21541/supp-9 Supplemental Information 9 STROBE-checklist

## Additional Information and Declarations

### Data Availability

The following information was supplied regarding data availability: The raw data is available in the Supplemental Files.
