# Supplementary figures and images for: Influence of vessel-depleted neck and risk factors on vascularized free flap failure: a retrospective cohort study and predictive model
Source: PeerJ. 2026 Jul 22;14:e21541. doi: 10.7717/peerj.21541 (PMC13401362; doi:10.7717/peerj.21541)

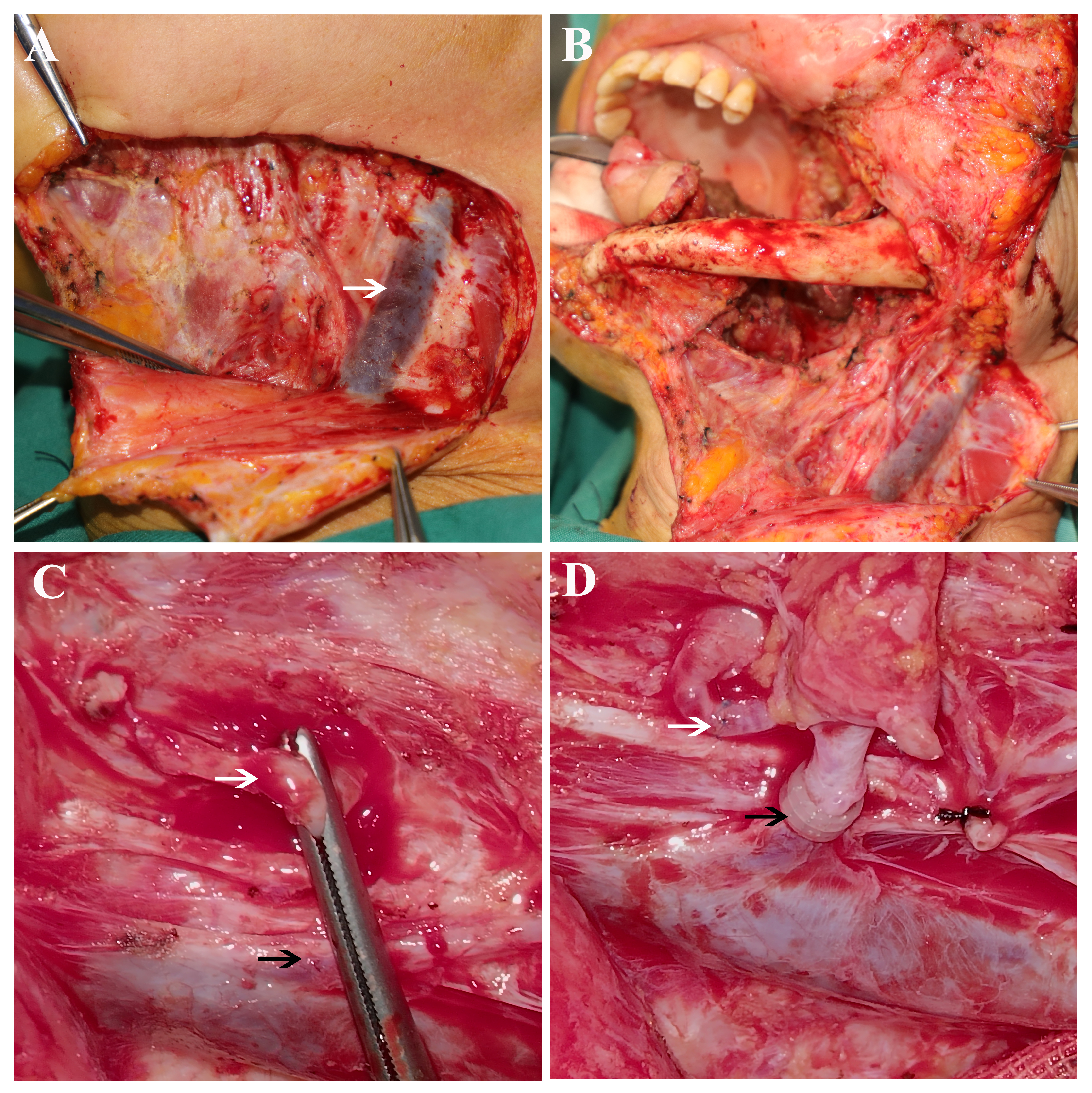

Supplement: Supplemental Information 1 — The presented patient was diagnosed as recurrence squamous cell carcinoma in tongue and has received left radical neck dissection with left anterior lateral thigh flap transplantation five years ago. This time the surgery plan was to use the right anterior medial thigh flap for reconstruction. (A) The initial exposure of left neck region. The left internal jugular vein was indicated (white arrow). (B) Resection of recurrent tongue lesion. (C) Microsurgical identification of recipient vessels: ipsilateral superior thyroid artery (white arrow) and internal jugular vein branch (black arrow). (D) End-to-end arterial anastomosis between superior thyroid artery and flap pedicle (white arrow). The venous anastomosis was applied between internal jugular vein branch and flap vein (black arrow). [file peerj-14-21541-s001.jpg]
