# Supplementary material for: Influence of vessel-depleted neck and risk factors on vascularized free flap failure: a retrospective cohort study and predictive model
Source: PeerJ. 2026 Jul 22;14:e21541. doi: 10.7717/peerj.21541 (PMC13401362; doi:10.7717/peerj.21541)
Supplement: Supplemental Information 2 [file peerj-14-21541-s002.docx]

Table S1. Characteristics of patients in training set based on vessel-depleted neck status.

|  | Vessel-depleted neck | |  |
| --- | --- | --- | --- |
| Characteristics | No (n=1445) | Yes (n=61) | P value |
| Age (SD) | 56.46 (15.073) | 56.52 (13.370) | 0.811 |
| Gender (%) |  |  | 0.252 |
| Female | 535(37.0) | 27(44.3) |  |
| Male | 910(63.0) | 34(55.7) |  |
| Smoking history |  |  | 0.518 |
| No | 864(59.8) | 39(63.9) |  |
| Yes | 581(40.2) | 22(36.1) |  |
| Alcohol intake history |  |  | 0.277 |
| No | 920(63.7) | 43(70.5) |  |
| Yes | 525(36.3) | 18(29.5) |  |
| Diabetes mellitus |  |  | 0.949 |
| No | 1283(88.8) | 54(88.5) |  |
| Yes | 162(11.2) | 7(11.5) |  |
| Hypertension |  |  | 0.248 |
| No | 1065(73.7) | 49(80.3) |  |
| Yes | 380(26.3) | 12(19.7) |  |
| Heart disease |  |  | 0.612^a^ |
| No | 1391(96.3) | 60(98.4) |  |
| Yes | 54(3.7) | 1(1.6) |  |
| Liver disease |  |  | 0.065^a^ |
| No | 1386(95.9) | 55(90.2) |  |
| Yes | 59(4.1) | 6(9.8) |  |
| Radiotherapy history |  |  | <0.001^a^ |
| No | 1397(96.7) | 31(50.8) |  |
| Yes | 48(3.3) | 30(49.2) |  |
| Chemotherapy history |  |  | <0.001^a^ |
| No | 1357(93.9) | 36(59.0) |  |
| Yes | 88(6.1) | 25(41.0) |  |
| Hospitalization history |  |  | <0.001 |
| No | 730(50.5) | 0(0) |  |
| Yes | 715(49.5) | 61(100) |  |
| Operation history |  |  | <0.001 |
| No | 798(55.2) | 0(0) |  |
| Yes | 647(44.8) | 61(100) |  |
| Recurrence |  |  | <0.001 |
| No | 1337(92.5) | 22(36.1) |  |
| Yes | 108 | 39(63.9) |  |
| Operation duration (minutes) | 342.89 (111.096) | 305.13 (89.064) | 0.011 |
| Blood loss (mL) | 345.52 (184.759) | 301.15 (135.697) | 0.033 |
| Tracheotomy |  |  | 0.974^a^ |
| No | 1364(94.4) | 57(93.4) |  |
| Yes | 81(5.6) | 4(6.6) |  |
| Titanium plate use |  |  | 0.012 |
| No | 807(55.8) | 44(72.1) |  |
| Yes | 638(44.2) | 17(27.9) |  |
| Disease location |  |  | - |
| Buccal | 331(22.9) | 16(26.2) |  |
| Floor of mouth | 134(9.3) | 5(8.2) |  |
| Gingiva | 173(12.0) | 5(8.2) |  |
| Mandible | 231(16.0) | 8(13.1) |  |
| Maxilla | 32(2.2) | 0(0) |  |
| Root of tongue | 59(4.1) | 3(4.9) |  |
| Soft palate/oropharynx | 64(4.4) | 5(8.2) |  |
| Tongue | 335(23.2) | 11(18.0) |  |
| Others | 86(5.9) | 8(13.1) |  |
| Disease types |  |  | 0.699 |
| Benign and others | 211(14.6) | 10(16.4) |  |
| Malignant | 1234(85.4) | 51(83.6) |  |
| Histopathology |  |  | 0.879 |
| Benign and others | 211(14.6) | 10(16.4) |  |
| I-III | 634(43.9) | 25(41.0) |  |
| IV | 600(41.5) | 26(42.6) |  |
| Flap types |  |  | 0.001^b^ |
| Anterolateral thigh flap | 1025(70.9) | 43(70.5) |  |
| Osteocutaneous flap | 219(15.1) | 3(4.9) |  |
| Lateral arm flap | 69(4.8) | 1(1.6) |  |
| Latissimus dorsi flap | 33(2.3) | 5(8.2) |  |
| Radial forearm free flap | 73(5.1) | 5(8.2) |  |
| Others | 26(1.8) | 4(6.6) |  |
| Artery anastomoses |  |  | 0.022^b^ |
| End-to-end | 1441(99.7) | 59(96.7) |  |
| End-to-side | 4(0.3) | 2(3.3) |  |
| Vein anastomoses |  |  | 0.505 |
| End-to-end | 1235(85.5) | 54(88.5) |  |
| End-to-side | 210(14.5) | 7(11.5) |  |

^a^, Continuity correction was used. ^b^, Fisher's exact test was used.
