# Supplementary material for: Influence of vessel-depleted neck and risk factors on vascularized free flap failure: a retrospective cohort study and predictive model
Source: PeerJ. 2026 Jul 22;14:e21541. doi: 10.7717/peerj.21541 (PMC13401362; doi:10.7717/peerj.21541)
Supplement: Supplemental Information 3 [file peerj-14-21541-s003.docx]

Table S2. Characteristics of patients in testing set based on vessel-depleted neck status.

|  | Vessel-depleted neck | |  |
| --- | --- | --- | --- |
| Characteristics | No (n=437) | Yes (n=22) | P value |
| Age (SD) | 57.56(14.446) | 59.09(11.191) | 0.749 |
| Gender (%) |  |  | 0.929 |
| Female | 163(37.2) | 8(36.4) |  |
| male | 274(62.7) | 14(63.6) |  |
| Smoking history |  |  | 0.389 |
| No | 259(59.3) | 11(50) |  |
| Yes | 178(40.7) | 11(50) |  |
| Alcohol intake history |  |  | 0.274 |
| No | 267(61.1) | 16(72.7) |  |
| Yes | 170(38.9) | 6(27.3) |  |
| Diabetes mellitus |  |  | 0.831^a^ |
| No | 380(87.0) | 20(90.9) |  |
| Yes | 57(13.0) | 2(9.1) |  |
| Hypertension |  |  | 0.655 |
| No | 298(68.2) | 14(63.6) |  |
| Yes | 139(31.8) | 8(36.4) |  |
| Heart disease |  |  | 1.000^a^ |
| No | 414(94.7) | 21(95.5) |  |
| Yes | 23(5.3) | 1(4.5) |  |
| Liver disease |  |  | 1.000^a^ |
| No | 416(95.2) | 21(95.5) |  |
| Yes | 21(4.8) | 1(4.5) |  |
| Radiotherapy history |  |  | <0.001^a^ |
| No | 420(96.1) | 10(45.5) |  |
| Yes | 17(3.9) | 12(54.5) |  |
| Chemotherapy history |  |  | <0.001^a^ |
| No | 399(91.3) | 11(50) |  |
| Yes | 38(8.7) | 11(50) |  |
| Hospitalization history |  |  | <0.001^a^ |
| No | 190(43.5) | 0(0) |  |
| Yes | 247(56.5) | 22(100) |  |
| Operation history |  |  | <0.001 |
| No | 196(44.9) | 0(0) |  |
| Yes | 241(55.1) | 22(100) |  |
| Recurrence |  |  | <0.001^a^ |
| No | 405(92.7) | 7(31.8) |  |
| Yes | 32(7.3) | 15(68.2) |  |
| Operation duration (minutes) | 310.03(99.552) | 322.41(132.527) | 0.999 |
| Blood loss (mL) | 278.03(127.676) | 225.45(117.178) | 0.023 |
| Tracheotomy |  |  | 0.105^a^ |
| No | 408(93.4) | 18(81.8) |  |
| Yes | 29(6.6) | 4(18.2) |  |
| Titanium plate use |  |  | 0.350 |
| No | 318(72.8) | 14(63.6) |  |
| Yes | 119(27.2) | 8(36.4) |  |
| Disease location |  |  | 0.380^b^ |
| Buccal | 112(25.6) | 8(36.4) |  |
| Floor of mouth | 36(8.2) | 0(0) |  |
| Gingiva | 60(13.7) | 4(18.2) |  |
| Mandible | 57(13.1) | 5(22.7) |  |
| Maxilla | 10(2.3) | 0(0) |  |
| Root of tongue | 14(3.2) | 0(0) |  |
| Soft palate/oropharynx | 14(3.2) | 1(4.5) |  |
| Tongue | 105(24.0) | 2(9.1) |  |
| Others | 29(6.7) | 2(9.1) |  |
| Disease types |  |  | 0.275^a^ |
| Benign and others | 54(12.4) | 5(22.7) |  |
| Malignant | 383(87.6) | 17(77.3) |  |
| Histopathology |  |  | 0.018 |
| Benign and others | 54(12.4) | 5(22.7) |  |
| I-III | 234(53.5) | 5(22.7) |  |
| IV | 149(34.1) | 12(54.5) |  |
| Flap types |  |  | 0.203^b^ |
| Anterolateral thigh flap | 335(76.7) | 18(81.8) |  |
| Osteocutaneous flap | 48(11.0) | 2(9.1) |  |
| Lateral arm flap | 9(2.1) | 1(4.5) |  |
| Latissimus dorsi flap | 4(0.9) | 1(4.5) |  |
| Radial forearm free flap | 40(0.9) | 0(0) |  |
| Others | 1(0.2) | 0(0) |  |
| Artery anastomoses |  |  | 0.179^b^ |
| End-to-end | 434(99.3) | 21(95.5) |  |
| End-to-side | 3(0.7) | 1(4.5) |  |
| Vein anastomoses |  |  | 0.942^a^ |
| End-to-end | 390(89.2) | 19(86.4) |  |
| End-to-side | 47(10.8) | 3(13.6) |  |
| Flap status |  |  | 1.000^b^ |
| Success | 422(96.6) | 22(100) |  |
| Failure | 15(3.4) | 0(0) |  |

^a^, Continuity correction was used. ^b^, Fisher's exact test was used.
