# Supplementary material for: Influence of vessel-depleted neck and risk factors on vascularized free flap failure: a retrospective cohort study and predictive model
Source: PeerJ. 2026 Jul 22;14:e21541. doi: 10.7717/peerj.21541 (PMC13401362; doi:10.7717/peerj.21541)
Supplement: Supplemental Information 4 [file peerj-14-21541-s004.docx]

Table S3. Univariate logistic regression of patient factors associated with flap failure in training set.

|  | Univariate Logistic Regression | |  |
| --- | --- | --- | --- |
| Parameters | OR (95%CI) | P value |  |
| Age | 0.976 (0.959-0.995) | **0.011** |  |
| Gender (Female/male) | 1.115 (0.590-2.106) | 0.738 |  |
| Smoking history (No/Yes) | 0.716 (0.375-1.367) | 0.312 |  |
| Alcohol intake history (No/Yes) | 1.052 (0.562-1.971) | 0.873 |  |
| Diabetes mellitus (No/Yes) | 0.379 (0.091-1.579) | 0.183 |  |
| Hypertension (No/Yes) | 1.103 (0.561-2.170) | 0.776 |  |
| Heart disease (No/Yes) | 1.298 (0.306-5.508) | 0.724 |  |
| Liver disease (No/Yes) | 3.859 (1.567-9.501) | **0.003** |  |
| Radiotherapy history (No/Yes) | 1.925 (0.670-5.530) | 0.224 |  |
| Chemotherapy history (No/Yes) | 1.274 (0.447-3.631) | 0.650 |  |
| Hospitalization history (No/Yes) | 1.317 (0.712-2.434) | 0.380 |  |
| Operation history (No/Yes) | 1.187 (0.647-2.177) | 0.580 |  |
| Recurrence (No/Yes) | 1.837 (0.803-4.206) | 0.150 |  |
| Vessel-depleted neck (No/Yes) | 1.817 (0.546-6.046) | 0.330 |  |
| Disease location |  |  |  |
| Buccal | 1 |  |  |
| Floor of mouth | 0.828 (0.221-3.106) | 0.780 |  |
| Gingiva | 1.767 (0.670-4.662) | 0.250 |  |
| Mandible | 1.470 (0.575-3.758) | 0.422 |  |
| Maxilla | 6.955 (2.177-22.215) | **0.001** |  |
| Root of tongue | 0.616 (0.077-4.947) | 0.648 |  |
| Soft palate/oropharynx | 0.552 (0.069-4.431) | 0.576 |  |
| Tongue | 0.551 (0.183-1.660) | 0.289 |  |
| Others | 0.816 (0.173-3.844) | 0.798 |  |
| Disease types (Benign/malignant) | 0.488 (0.242-0.982) | **0.044** |  |

Bolded values indicate statistical significance.

OR, odds ratio. CI: confidence interval.
