# Supplementary material for: Influence of vessel-depleted neck and risk factors on vascularized free flap failure: a retrospective cohort study and predictive model
Source: PeerJ. 2026 Jul 22;14:e21541. doi: 10.7717/peerj.21541 (PMC13401362; doi:10.7717/peerj.21541)
Supplement: Supplemental Information 5 [file peerj-14-21541-s005.docx]

Table S4. Univariate logistic regression of surgical factors associated with flap failure in training set.

|  | Univariate Logistic Regression | |  |
| --- | --- | --- | --- |
| Parameters | OR (95%CI) | P value |  |
| Operation duration (minutes) | 1.002 (0.999-1.005) | 0.117 |  |
| Blood loss (mL) | 1.000 (0.998-1.002) | 0.912 |  |
| Tracheotomy use (No/Yes) | 1.263 (0.383-4.169) | 0.701 |  |
| Titanium plate use (No/Yes) | 2.244 (1.199-4.200) | **0.012** |  |
| Number of microanastomosis |  |  |  |
| 1 artery 1 vein | 1 |  |  |
| 1 artery 2 veins | 0.835 (0.452-1.543) | 0.565 |  |
| 2 arteries 2 veins | 0 | 1.000 |  |
| Surgical site infection (No/Yes) | 7.400 (2.415-22.672) | **<0.001** |  |
| Flap types |  |  |  |
| Anterolateral thigh flap | 1 |  |  |
| Osteocutaneous flap | 1.462 (0.684-3.124) | 0.327 |  |
| Lateral arm flap | 0 | 0.997 |  |
| Latissimus dorsi flap | 2.966 (0.864-10.184) | 0.084 |  |
| Radial forearm free flap | 0.449 (0.060-3.339) | 0.434 |  |
| Others | 0 | 0.998 |  |
| Facial artery use (No/Yes) | 1.754 (0.774-3.974) | 0.178 |  |
| Artery anastomosis (end-to-end/end-to-side) | 0 | 0.999 |  |
| Internal jugular vein use (No/Yes) | 0.545 (0.282-1.053) | 0.071 |  |
| Facial vein use (No/Yes) | 1.857 (0.997-3.460) | 0.051 |  |
| Vein anastomosis (end-to-end/end-to-side) | 0.962 (0.401-2.308) | 0.931 |  |

Bolded values indicate statistical significance.

OR, odds ratio. CI: confidence interval.
