# Supplementary material for: Influence of vessel-depleted neck and risk factors on vascularized free flap failure: a retrospective cohort study and predictive model
Source: PeerJ. 2026 Jul 22;14:e21541. doi: 10.7717/peerj.21541 (PMC13401362; doi:10.7717/peerj.21541)
Supplement: Supplemental Information 6 [file peerj-14-21541-s006.docx]

Table S5. Arteries selection of vessel-depleted neck cases in two sets.

|  | Training set (61) | | Testing set (22) | |
| --- | --- | --- | --- | --- |
| Artery | Contralateral (39) | Ipsilateral (22) | Contralateral (11) | Ipsilateral (11) |
| Contralateral facial artery | 33 |  | 9 |  |
| Contralateral submental artery | 2 |  | 0 |  |
| Contralateral superior thyroid artery | 4 |  | 2 |  |
|  |  |  |  |  |
| Artery of previous flap |  | 2 |  | 0 |
| Ipsilateral external carotid artery |  | 2 |  | 1 |
| Ipsilateral facial artery |  | 1 |  | 0 |
| Ipsilateral lingual artery |  | 1 |  | 0 |
| Ipsilateral occipital artery |  | 1 |  | 0 |
| Ipsilateral superficial temporal artery |  | 8 |  | 4 |
| Ipsilateral superior thyroid artery |  | 7 |  | 4 |
| Ipsilateral maxilla artery |  | 0 |  | 1 |
| Ipsilateral supraclavicular artery |  | 0 |  | 1 |
